# Biased sampling activity: an investigation to promote discussion

**DOI:** 10.1111/test.12165

**Published:** 2018-06-20

**Authors:** Simon R. White, Laura J. Bonnett

**Affiliations:** ^1^ MRC Biostatistics Unit University of Cambridge Cambridge UK; ^2^ Department of Biostatistics University of Liverpool Liverpool UK

**Keywords:** Teaching, Teaching statistics, Sampling, Biased sampling, Practical activity

## Abstract

The statistical concept of sampling is often given little direct attention, typically reduced to the mantra “take a random sample”. This low resource and adaptable activity demonstrates sampling and explores issues that arise due to biased sampling.

## Introduction

It is difficult to communicate statistical concepts in a meaningful way using only abstract definitions. Using interactive activities that highlight the key concept, consisting of a physical process that can be followed and manipulated, can greatly support learning. The focus of this article is to describe such an activity to demonstrate sampling.

Sampling is an interesting concept to communicate. With a reasoned line of questions, we can convey an intuitive understanding of the concept in genuine population contexts, namely, that ‘taking a sample’ is required to learn something about a large group without having to investigate everyone. By large group, we mean the population of interest, which naturally leads to a discussion about the term *population*; differentiating its common meaning – number of living things or items – from its traditional statistical usage covering all contexts and meaning a general situation described by a probability model.

There are many questions we can use to stimulate this discussion and give it a real‐life context. For example, ‘what pets do people own?’, ‘how many people have dementia?’, ‘how will people vote in the next election?’ or ‘in how many films does the lead actress have more lines than the lead actor?’ In each case, we first have to consider what the population of interest is; for questions about people, we may consider a local region, a country or even all the people of the world, but that does not make sense for how people will vote in an election – with different political parties in different countries. When thinking about films, we have to think about what we mean by the word ‘film’: only films with a lead actress and lead actor – not all films have both, only fictional films or documentaries, major studio releases or fan‐made short films, all films ever or only in recent years, what about different languages. This may seem pedantic, but it can be at the core of a discussion on sampling.

If we could find every member of the population, then we would find *the* answer to our question, there would be no uncertainty. However, i.e. typically not feasible (either it would take too long or cost too much money) so instead we only consider some members of the population – we take a sample. From a sample, we will obtain an estimate of a quantity of interest about the population.

The aim of this activity is to explore ideas around taking a sample, with a focus on the issue of biased sampling. The learning aim is to understand the need for a well‐designed sampling scheme to ensure an unbiased sample from the population that will lead to an unbiased estimate.

## Overview

The activity is designed in such a way that participants will typically generate nonrandom samples, i.e. the probability of a given sample is not equal to the probability of drawing the individuals at random. We will call samples generated in this way biased samples; since this will result in estimates that are quickly shown to be biased.

The scope and depth of the investigation can be tailored to the available time, context of the activity and level of the target audience by including different aspects. In the following sections, we present a suggested template for delivering the activity and key discussion points.

As an aside, especially if using this as a classroom investigation, it may be appropriate to discuss how to use weighing scales properly. Specifically, ensuring that the scales are set to zero with the container in place, using the tare feature to zero the scales.

## Materials

**Figure 1:**
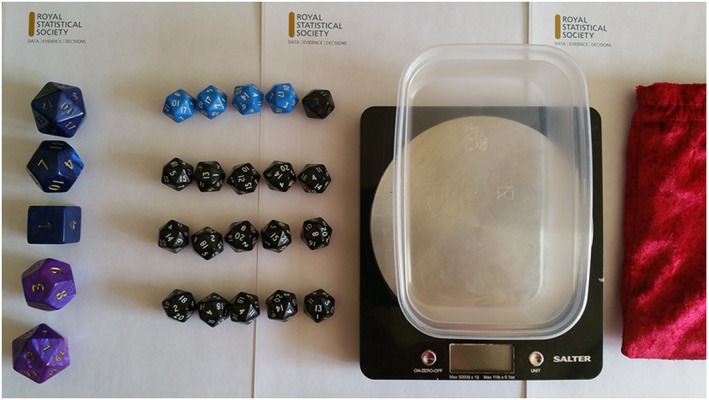
Example of required materials: 25 objects, a weighing scale and a draw bag. Note that we have small/large and blue/not‐blue objects. [Colour figure can be viewed at wileyonlinelibrary.com]

The required resources are minimal and easy to source or adapt, making the activity portable for science festivals, as well as reasonable to create multiple sets for use in a classroom investigation. Figure 1 illustrates an example set of materials using dice; we have also successfully used chocolate bars (from a well‐known company) that come in full‐sized and miniature‐sized versions, building blocks (wooden and plastic) and other toys.

Our set requires 25 objects of two distinct sizes distinguishable using a visual trait (e.g. colour). It is important that the different sized objects, which we shall call small and large, have distinct weights and are easy to distinguish by touch. For the visual trait, we shall use colour and the labels blue and not‐blue. Hence, the four types of object are small–blue, large–blue, small–not‐blue and large–not‐blue. The exact number of each type does not matter, however, we have found a good mix to be 3 large–blue, 2 large–not‐blue, 4 small–blue and 16 small–not‐blue (figure 1). In a classroom setting, the multiple sets do not need to be identical.

In addition to the objects, we require a set of weighing scales and a bag able to contain all the objects and easily draw out samples. Note, it is important that the small objects are sufficiently heavy to register individually on the scales. We recommend small objects weighing 5–10 g and large objects weighing 20–50 g.

## Weighing Activity

### Determine the mean object weight

To prepare for the activity, place the objects in the bag in secret, then explain the problem: there are 25 objects in the bag and we are interested in their total weight, how could we work that out?

Explain that the trivial solution to weigh all the objects is often not possible. Instead, if we knew the mean weight of an object, we could calculate the weight of the bag as 25 times the mean weight. Hence, the weight of the bag is equivalent to knowing the mean object weight. There is no prior knowledge about the objects, so we will take a sample of objects and use these to estimate the population mean object weight; here, we introduce the concept of a sample mean (or fully, the sample mean weight).

### Defining unbiased mean

It must be stressed that there are lots of different possible samples, each will (likely) result in a different sample mean. A desirable property of a sample mean would be that, across all possible samples, we would expect the sample mean to equal the (true) population mean.

Informally, if we imagine all possible samples we could take, then the mean of all the associated sample means would equal the population mean. We call such estimates unbiased. Formally, let *M* denote the population mean and *m*_*k*_ denote the sample mean from sample *k*, then we are saying that the expected value or mean of the sample means being equal to the population mean, E_*k*_[*m*] = *M*, is a desirable property and called being unbiased.

As an aside, typical notation for the population mean would be the Greek letter *μ*, but this may confuse presenting the activity. Further, this activity can be presented entirely verbally without relying on any writing board/flipchart/etc. Hence, in this article, we present a written/notation‐based and descriptive approach to explaining the activity.

In summary, we do not expect a specific sample mean to exactly equal the population mean, i.e. the ‘price’ of using a sample – you cannot get something for nothing. However, we expect that ‘on average’ across all possible samples, we would obtain the population mean.

### Activity

Instruct the participant to draw a sample of objects at random from the bag, weigh this sample, calculate the sample mean (i.e. add all the weights and divide by the number of objects) and hence obtain an estimate of the total weight of the bag. With 25 objects as described in the Materials section, use a sample size equal to the number of large objects, namely, a sample size of five (this will be helpful when revealing the population and samples in the next stage).

The participant may realize there are different types of object within the bag. If they ask or mention this while drawing their sample, remind them to draw a random sample from the bag.

At this point, it may be worth exploring the use of the mean object weight in the presence of different types of object. We are interested in the total weight of the population, *W*, that consists of *N* objects. Hence, the population mean object weight is *M* = *W*/*N*. It does not matter that the *N* objects may have different weights. We collect a sample to estimate the population mean object weight, *M*, with our sample mean *m*, so we are explicitly allowing for objects to have different weights.

The participant should then repeat the activity, replacing all the objects and drawing a new sample (of the same size, five say) and recording the new sample mean. Let the participant have several repeats, each will likely have a different estimate.

**Figure 2:**
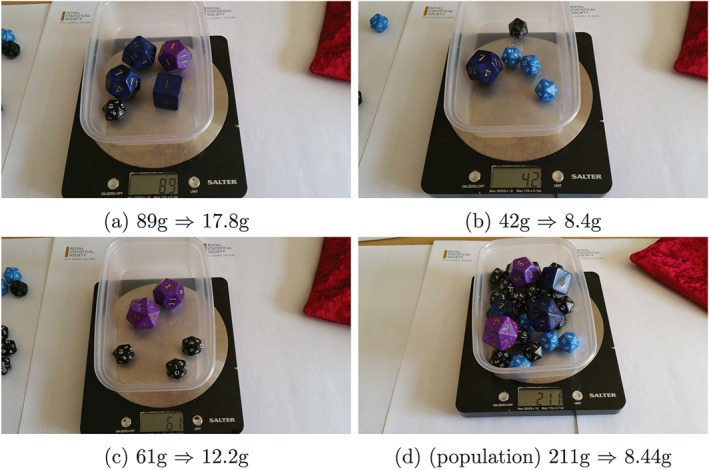
The mean object weight from three samples of five objects (a–c) and the true population mean object weight (d). [Colour figure can be viewed at wileyonlinelibrary.com]

For example, in figure 2, the participant's first estimate of the mean object weight is 17.8 g, then 8.4 and 12.2 g. The true population mean object weight is 8.44 g.

### Reveal the population and possible samples

Empty the entire bag but do not weigh all the objects just yet. Following from figure 1 (and Materials section), there will be 5 large and 20 small objects. The participant should recognize some of the objects from their samples, we hope each sample contained some unique objects but that is not necessary.

Return the discussion to the different possible samples, for the moment, ignore the slight variation in weight between similar objects and say we have large (L) objects that each weigh *w*_*L*_ and small (S) objects that each weigh *w*_*S*_.

Continuing our example, if we take a sample of size five, how many different types of sample can we have? The answer is six, specifically: (LLLLL), (LLLLS), (LLLSS), (LLSSS), (LSSSS) and (SSSSS). The order of drawing objects from the bag does not matter. Ask the participant to recall what their samples consisted of, how many of these six types of sample did they draw?

Although there are six different types of sample, explain that they do not occur equally often if drawing objects at random. We can calculate the number of ways of drawing *l* large and *s* small objects. Given the sample size, we can work out how many possible random samples of each type there are, essentially the number of ways of choosing the objects from the bag. The formula involves the product of two binomials,

#ls=LlSs=L!L−l!l!S!S−s!s!,

$$\text{where}\;a!=\left(a\right)\times \left(a-1\right)\times \left(a-2\right)\times \cdot \cdot \cdot \times \left(2\right)\times \left(1\right).$$

When *n* = 25, we have the following table of the number of different samples of size five:

**Table:** 

#{5,0}	LLLLL	1
#{4,1}	LLLLS	100
#{3,2}	LLLSS	1,900
#{2,3}	LLSSS	11,400
#{1,4}	LSSSS	24,225
#{0,5}	SSSSS	15,504

It is not necessary to work out these numbers exactly with the participant. If the sample size equals the number of large objects (which we recommend), then there is only one possible sample consisting only of large objects – i.e. the starting point. Then, consider the number of ways to draw one small and the remainder large objects, which can be seen to be *L* × *S* by picking one of the *L* large objects to omit and one of the *S* small objects to replace it. It is usually sufficient to explain the first and second lines in the above table.

The important point to make is that if we take the ‘average’ sample mean across all these possible samples, it will equal the population mean (shown in algebra in Appendix A) assuming that all of the 53,130 ( =255) possible samples are equally likely; which is true under random sampling.

### Discussion and biased estimate

With the participant's estimates to hand, weigh all the objects and compare; in our example (figure 2), the population mean weight is 8.44 g. Recall, we do not expect any estimate to be exactly right, i.e. the ‘price’ of taking a sample. However, to be a useful estimate, we would like it to be unbiased, as previously described in the Defining unbiased mean section.

At this point, reveal a chart of all previous participant's first estimate. Although participants may have multiple repeats, only record the first on this overall chart. Consider the distribution of estimates, ‘on average’ do they match the true total weight of all objects?

In our experience running this activity, and encouraged by the design of the activity, samples will typically include more of the large objects than they should; this will result in an over estimate, a biased estimate, of the mean weight (and hence the total weight).

It is essential that this part of the activity is not mentioned before the participant has drawn their first sample. The activity is designed to induce biased samples based on a natural inclination to grab larger objects within the bag. If the participant is aware of this effect, they will likely consciously alter their sampling behaviour.

If the activity is ‘working’, then participants' first guesses will give a distribution of sample means such that the expected value will be larger than the population mean – on average, participants will overestimate the sample mean. That is, we will have a biased estimate of the population mean.

The exact bias is difficult to understand using algebra, different participants may have a different sampling bias (including no bias at all). The effect of taking a biased sample is that all the possible samples are no longer equally likely; but not every participant may be biased in the same way. An unfortunate outcome might be that bias in both directions is present, one group of people tend to oversample the large objects while another tend to oversample the small objects.

At the end of the activity, it is important to return to the original example/context used to motivate the activity.

### How much should we ‘trust’ an estimate? A question of confidence

When taking a single sample, even if it is an unbiased random sample, there is uncertainty in the estimated mean. So an obvious question to explore is how much do we ‘trust’, or what is our confidence in, the estimated mean?

Firstly, what do we mean by trust or confidence? We know that, by chance, we may get an estimate that is quite different to the population mean. But how likely is that? For any given sample mean, can we say something about how confident we are in that estimate of the population mean?

For advanced groups, this discussion naturally leads into the topics of standard deviation, standard errors and confidence intervals. However, that goes beyond the scope of this article. Instead of explicitly calculating the confidence interval, we could present example uncertainties. For example, we may consider the impact of uncertainty when the mean weight estimate is 10 g plus or minus 10 g, compared with 10 g plus or minus 1 g; these two ranges of uncertainty change our opinion (or more colloquially, our trust) of the 10 g estimate.

## Proportion Activity

After considering estimates of weights in the Weighing Activity section, we translate the key learning points to a different context; namely, estimating the proportion of objects that are blue by taking a sample of 10 objects.

The activity follows exactly as before, except we record the proportion of blue objects. If continuing from sample means the participant will be familiar with the issue of biased sampling. Swapping object sets, so that the bag contains a new set of unknown objects would effectively reset the problem. However, it would not alter the knowledge gained about sampling; it is likely participants will now consciously alter their sampling behaviour. That is worth mentioning explicitly, as the purpose of this activity is to consider if we can have biased proportions in the same way we had biased means.

### Possible estimates

Why did we change the sample size from 5 to 10 for estimating a proportion? With a sample of five objects, the only possible proportion estimates are 0%, 20%, 40%, 60%, 80% or 100%. Unlike when investigating the mean object weight, the range of possible proportion estimates is directly linked to the sample size.

The population proportion in our example (figure 1) is 7/25 = 28%. Hence, without a larger sample size, we cannot obtain estimates that are close. As an extreme example, imagine a sample of only one object, then our estimated proportion can be either 0% or 100%.

Recall that we do not expect the sample estimate to exactly equal the population value. The case of proportions clearly demonstrates this, since it is highly unlikely that the set of possible sample proportions includes the true population proportion; as is the case in our activity example. However, this does not impact our concept of an unbiased estimate. Although no sample estimate may equal the truth, the average of all possible sample estimates may.

### Is bias a problem for proportions?

When weighing the objects, there were distinct weights for small and large objects, this in turn led to a biased estimate of the mean when a biased sample is taken. Although not as obvious, there is a difference with regard to the colour, 60% (3/5) of the large objects are blue compared 20% (4/20) of the small objects. A biased sample that oversamples the large objects will overestimate the proportion of blue objects, similarly to over estimating the weight. This is a subtle point, translating the obvious difference in object weight to a difference in proportion of blue objects by size.

To extend this idea, and to challenge understanding of biased sampling, imagine a bag where the proportion of blue objects was the same across small and large objects; in this case, would a sample biased due to object size matter when estimating the proportion? The answer is no, because even if we over represent the large objects, they are equivalent to the small in terms of colour.

## Summary

The concept of sampling is often discussed in abstract terms, with the message that most people learn being ‘take a random sample’ and everything will be fine. We have presented a tried and tested activity that is designed to explore the statistical ideas of sampling in a practical way, specifically demonstrating the issue of biased sampling.

We have used the activity at several science festivals, where there is a strong need to adapt the content based on the age and level of the audience; alternatively, it can be part of a classroom investigation and used as a foundation to introduce more advanced topics, such as quantifying uncertainty and confidence intervals.

Designing good studies is the cornerstone of good science. There are numerous recent and historical examples in political science of biased samples; see Kennedy et al. (2016) for an example of modern election polling and Rothman (2016) for an account of the Literary Digest's infamous 1936 poll. It is not only political science that suffers from biased sampling and examples can be found in many contexts. Any situation where the make‐up of the sample is affected by another factor can induce sampling bias, e.g. the so‐called survivorship‐bias demonstrated by Abraham Wald when studying damage to aircraft; nicely summarized by Jordan Ellenberg (2016). This can also lead into discussions on nonrandom samples, e.g. convenience samples; in the context of our activity if it is easier (more convenient) to sample the large objects, they will appear in samples more often than by chance.

The key message of the activity is to explore the idea of bias in sampling. The aim of the activity is for participants to gain a deeper insight and understanding of the overly simple and overused mantra ‘take a random sample’, and the potential impact on estimates if the mantra is not heeded.

## Appendix A Proof of unbiasedness

### A.1

Using the following algebra, we can show that, given a random sample where all possible samples are equally likely, we obtain an unbiased estimate of the mean object weight.

Recall that to be unbiased, the average of all possible sample mean weights, *m*_*k*_, should be equal to the true population mean weight, *M*. From the table in the Reveal the population and possible samples section, we know how many different ways there are to draw a sample consisting of a number of small and large objects, with an associated sample mean weight. So we take the average of all those possible samples, one term per row in the table,

$$E_{k}\left[m\right]=\frac{1}{53130}\left[\left(1)(\frac{5w_{L}}{5}\right)+\left(100)(\frac{4w_{L}+w_{S}}{5}\right)+\left(1900\right)\left(\frac{3w_{L}+2w_{S}}{5}\right)+\left(11400\right)\left(\frac{2w_{L}+3w_{S}}{5}\right)+\left(24225\right)\left(\frac{w_{L}+4w_{S}}{5}\right)+\left(15504\right)\left(\frac{5w_{S}}{5}\right)\right]=\frac{1}{53130}\left[\frac{53130w_{L}+212520w_{S}}{5}\right]=\frac{w_{L}+4w_{S}}{5}$$

which can also be written as,

$$=\frac{5w_{L}+20w_{S}}{25}\;\mathrm{by}\;\text{definition this is},=M.$$

Hence, the average of all possible sample mean weights equals the population mean weight.
